# Cardiotrophin-1 Induces Matrix Metalloproteinase-1 in Human Aortic Endothelial Cells

**DOI:** 10.1371/journal.pone.0068801

**Published:** 2013-07-23

**Authors:** Akinori Tokito, Michihisa Jougasaki, Tomoko Ichiki, Shuichi Hamasaki

**Affiliations:** 1 Institute for Clinical Research, National Hospital Organization Kagoshima Medical Center, Kagoshima, Japan; 2 Department of Cardiovascular, Respiratory and Metabolic Medicine, Graduate School of Medicine, Kagoshima University, Sakuragaoka, Kagoshima, Japan; Brigham and Women's Hospital, Harvard Medical School, United States of America

## Abstract

Rupture of an atherosclerotic plaque is a key event in the development of cardiovascular disorders, in which matrix metalloproteinase-1 (MMP-1) plays a crucial role by degradation of extracellular matrix resulting in plaque instability. Cardiotrophin-1 (CT-1), a member of interleukin-6-type proinflammatory cytokines, has potent cardiovascular actions and is highly expressed in vascular endothelium, however its role in atherosclerosis has not been fully elucidated to date. The present study was designed to investigate whether CT-1 induces MMP-1 in human aortic endothelial cells (HAECs). Ribonuclease protection assay demonstrated that MMP-1 gene level in HAECs was enhanced by the treatment of CT-1 in a dose- and time-dependent manner. Immunocytochemical staining, Western immunoblot analysis and enzyme-linked immunosorbent assay revealed that CT-1 augmented MMP-1 protein synthesis and secretion. MMP-1 activity assay revealed that MMP-1 present in the supernatant of HAECs was exclusively precursor form. Casein zymography disclosed proteolytic activity in the supernatant of HAECs, which was enhanced by CT-1 treatment. Furthermore, pharmacological inhibitor study indicated the important roles of extracellular signal-regulated kinase (ERK) 1/2, p38 mitogen-activated protein (MAP) kinase, c-Jun N-terminal kinase (JNK) and Janus kinase/signal transducers and activators of transcription (JAK/STAT) signaling pathways in mediating CT-1-induced MMP-1 gene and protein expression. These data reveal for the first time that CT-1 induces the proteolytic potential in HAECs by upregulating MMP-1 expression through ERK1/2, p38 MAP kinase, JNK and JAK/STAT pathways, and suggest that CT-1 may play an important role in the pathophysiology of atherosclerosis and plaque instability.

## Introduction

Atherosclerosis, the underlying condition of acute coronary syndrome and stroke, is widely recognized as an inflammatory disease in which various cytokines and growth factors are involved [1]. In later stages of this disease, rupture of an atherosclerotic plaque and subsequent thrombosis are the key events in development of cardiovascular disorders [2]. The rupture of a plaque is largely based on the instability of the plaque which is rich in cholesterol, and the structural vulnerability caused by alteration between accumulation and degradation of extracellular matrices (ECM).

Matrix metalloproteinases (MMPs) are a superfamily of zinc-dependent endopeptidases that play a central role in the maintenance and remodeling of ECM. A large body of evidence implicates MMPs in all stages of atherosclerosis [3]. Elevated activities of MMPs in atherosclerotic lesions, caused either by up-regulation of their expression or down-regulation of their cognate inhibitors, have been implicated in plaque instability and eventually in rupture [2], [4]. Among this superfamily, MMP-1 (interstitial collagenase) is a key enzyme to cleave native fibrillar collagens, especially type I and III, providing the biochemical strength for fibrous plaques [5]. In addition, previous studies demonstrated that MMP-1 expression and activity are increased in advanced and unstable atherosclerotic plaques [6], [7]. To date, a lot of proinflammatory mediators or cytokines, such as angiotensin II, C-reactive protein, vascular endothelial growth factor, tumor necrosis factor-α and interleukin (IL)-1α have been proven to induce MMP-1 expression in vascular cells [8]–[11].

Cardiotrophin-1 (CT-1) is one of the proinflammatory cytokines belonging to the IL-6 cytokine family that includes IL-6, IL-11, IL-27, leukemia inhibitory factor (LIF), oncostatin M, cardiotrophin-like cytokine, ciliary neurotrophic factor and neuropoietin. The IL-6 cytokine family exerts biological actions through homodimerization or heterodimerizaiton of glycoprotein (gp)130, which subsequently activate signal transduction pathways, such as Ras-dependent mitogen-activated protein (MAP) kinase pathway and Janus kinase (JAK)/signal transducers and activators of transcription (STAT) cascade [12], [13]. This family exhibits multiple effects *in vivo* by modulating inflammatory and immunological processes [14], and CT-1 also has a broad spectrum of biological activities including cardiovascular actions [15]. However, it has not been fully elucidated whether CT-1 affects atherosclerosis. We previously showed that CT-1 stimulates expression of endothelin-1, a vasocontricting peptide, in vascular endothelial cells [16]. More recently, we also demonstrated that CT-1 and its receptor components are colocalized in human atherosclerotic plaque and that CT-1 enhances monocyte adhesion and migration by stimulating intercellular adhesion molecule-1 and monocyte chemoattractant protein-1 (MCP-1) [17]. These data support the possibility that CT-1 plays a crucial role in the pathophysiology of vascular inflammation and atherosclerosis. Additionally, Talwar et al. [18] reported that plasma concentration of CT-1 is elevated in patients with unstable angina compared with those with stable angina, providing the hypothesis that CT-1 is associated with increased susceptibility to plaque instability and rupture. As far as we know, few studies have ever showed a correlation between IL-6 cytokine family and MMP-1 in vascular cells [19]. Therefore, to extend our knowledge of the role of CT-1 in the vascular disorders, the current study was designed to identify the effect of CT-1 on expression of MMP-1 in human aortic endothelial cells (HAECs). In addition, we clarified the mechanisms involved in the regulation of MMP-1 expression by CT-1.

## Materials and Methods

### Reagents

Recombinant human CT-1 was purchased from PeproTech (Rocky Hill, USA). The mouse monoclonal anti-human MMP-1 antibody for immunohistochemistry and Western immunoblot analysis was from Daiichi Fine Chemical (Toyama, Japan), and that for neutralization of MMP-1 in zymography was from R&D systems (Minneapolis, USA). The mouse monoclonal antibody against β-actin was from Santa Cruz Biotechnology (Santa Cruz, USA). The goat polyclonal antibodies against human Toll-like receptor 4 (TLR4) and MCP-1, the mouse monoclonal antibodies against human gp130, LIF receptor (LIFR), CT-1 and IL-6 and the normal mouse IgG were from R&D Systems (Minneapolis, USA). The rabbit monoclonal antibodies specific for JAK1, JAK2, phospho-JAK2 (Tyr1007/Tyr1008) and c-Jun N-terminal kinase (JNK) were from Cell Signaling Technology (Beverly, USA). The rabbit monoclonal antibody specific for phospho-JAK1 (Tyr1022/Tyr1023) was from Life Technologies (Carlsbad, USA). The rabbit polyclonal antibodies specific for STAT1, phospho-STAT1 (Tyr701), STAT3, phospho-STAT3 (Tyr705) and phospho-JNK (Thr183/Tyr185) were from Cell Signaling Technology (Beverly, USA). Pharmacological inhibitors, such as PD-98059, SB-203580 and AG490 were obtained from Wako Pure Chemical (Osaka, Japan). SP-600125 was from BIOMOL (Plymouth Meeting, USA). JAK3 inhibitor II and piceatannol were from Calbiochem (La Jolla, USA).

### Cell culture of HAECs

HAECs (Lonza, Walkersville, USA) were seeded in plastic plates precoated with type I collagen (Asahi Techniglass, Nagoya, Japan) and maintained in medium 199 (Life Technologies, Carlsbad, USA) supplemented with 10% heat-inactivated fetal calf serum, 0.5 μg/mL fungizone, 0.25 μg/mL amphotericin B, 100 μg/mL streptomycin, 100 U/mL penicillin (Life Technologies, Carlsbad, USA), 14 U/mL heparin (Ajinomoto, Tokyo, Japan), 20 μg/mL endothelial cell growth supplement (Kohjin Bio, Saitama, Japan) and 10 μg/mL human epidermal growth factor (PeproTech, Rocky Hill, USA). HAECs were cultured at 37°C in 5% CO_2_ and 95% air in a humidified atmosphere. At confluence, HAECs formed a typical “cobblestone” monolayer. HAECs in the third to sixth passages were used in the present experiments.

### Total RNA extraction and ribonuclease protection assay (RPA)

Total RNA was extracted from HAECs using Pure Link RNA Mini kit (Ambion, Austin, USA). A biotin-labeled antisense RNA probe cocktail was transcribed from a set of custom-designed cDNA templates (BD Biosciences Pharmingen, San jose, USA) using MAXIscript in vitro transcription kit (Ambion, Austin, USA). Full-length sizes of probes for MMP-1 and glyceraldehyde-3-phosphate dehydrogenase (GAPDH) were 309 and 124 bp, respectively, and protected fragment sizes were 280 and 96 bp, respectively. The biotin-labeled antisense probes were hybridized to 5 µg of total RNA and subjected to ribonuclease digestion with RPAIII kit (Ambion, Austin, USA). The ribonuclease-protected fragments were purified, resolved on 6% denaturing tris-borate-EDTA-urea polyacrylamide gels (Life Technologies, Carlsbad, USA) and transferred to nylon membranes (Life Technologies, Carlsbad, USA). The protected fragments were visualized by incubation of the membranes with an alkaline phosphate-streptavidin solution with BrightStar BioDetect chemiluminescence reagent (Ambion, Austin, USA). The intensities of the blots of MMP-1 mRNA were quantified using a LAS-3000 Lumino image analyzer (Fuji Film, Tokyo, Japan) and normalized to GAPDH mRNA.

### Immunocytochemistry

Cultured HAECs were immediately fixed with 1% buffered paraformaldehyde (Wako Pure Chemical, Osaka, Japan) for 20 min. The indirect immunoperoxidase method was used for immunocytochemical analysis, as described previously [20]. The primary antibody against MMP-1 was used at 100-fold dilution. The specificity of the immunostaining was confirmed by substitution of the normal mouse IgG for primary antibody.

### Western immunoblot analysis

HAECs were washed with cold PBS and immediately harvested in ice-cold cell lysis buffer (Cell Signaling Technology, Beverly, USA) together with 1 mmol/L phenylmethylsulfonyl fluoride (Roche Diagnostics, Mannheim, Germany) and protease inhibitor cocktail, Complete Mini (Roche Diagnostics, Mannheim, Germany). Aliquots of 10 µg of proteins were resuspended in 3× SDS sample buffer (6% SDS, 30% glycerol, 0.03% bromphenol blue, 187.5 mmol/L Tris-HCl) and 1.25 mol/L Dithiothreitol (BioLabs, Ipswich, USA), sonicated, boiled for 5 min, and separated by 4–12% NuPAGE Bis-Tris gels (Life Technologies, Carlsbad, USA). The proteins were transferred to a polyvinylidene difluoride membrane (Life Technologies, Carlsbad, USA) by electroblotting for 2 hours. The membrane was soaked in blocking buffer (5% nonfat dry milk in 0.1% Tween-TBS) and then incubated with the primary phospho-specific antibodies or the primary antibody against MMP-1 at concentrations suggested by the manufactures in 5% BSA and 0.1% Tween-TBS overnight at 4°C. After washing in 0.1% Tween-TBS, the membrane was incubated with alkaline phosphate-conjugated secondary antibodies (Cell Signaling Technology, Beverly, USA) for 1 hour. The protein bands were visualized by CDP-Star chemiluminescent substrate (Cell Signaling Technology, Beverly, USA). The intensities of the blots were quantified using a LAS-3000 Lumino image analyzer (Fuji Film, Tokyo, Japan). The blots were stripped and reprobed with respective total antibodies or primary antibody against β-actin.

### Measurement of MMP-1 protein in the supernatant

Concentration of total MMP-1 protein (precursor form and active form) in supernatant was determined by using a commercially available enzyme-linked immunosorbent assay (ELISA) kit according to the manufacturer's instruction (Daiichi Fine Chemical, Toyama, Japan). Briefly, after incubation, the supernatant from HAECs was removed and applied at 100-fold dilution. The diluted samples and standards were added to each well of microtiter plate, which was precoated with anti-human MMP-1 monoclonal antibodies, and incubated for 1 hour. Each well was washed and incubated with enzyme-linked monoclonal antibodies specific for human MMP-1 for 30 min. The wells were washed to remove unbound antibody-enzyme reagent, and substrate solution was added to each well. After incubation for 30 min at room temperature, the enzyme reaction was stopped. MMP-1 concentrations were determined by comparison of the optical density results with the standard curve. Variations of intra- and inter-assay were 7% and 6%, respectively.

Similarly, another ELISA kit (R&D Systems, Minneapolis, USA) which is designed to evaluate both total and active form of MMP-1 in culture supernatant was performed according to the manufacture's instructions. Total MMP-1 level was measured by additional incubation with *p*-aminophenylmercuric acetate (APMA) to activate the precursor form of MMP-1. The relative fluorescence units were measured using a fluorescence plate reader, Infinite 200 PRO (TECAN, Kanagawa, Japan).

### Measurement of proteolytic activity

Proteolytic activity was examined using zymogram gels with a casein substrate as previously reported with some modifications [21]. Briefly, aliquots of supernatant from HAECs, with and without CT-1 treatment, were mixed with 2× Tris-Glycine SDS sample buffer (Life Technologies, Carlsbad, USA), loaded on a 12% Tris-Glycine gel with 0.05% casein (Life Technologies, Carlsbad, USA) and run for 90 min. After electrophoresis, the gel was treated with renaturing buffer (Life Technologies, Carlsbad, USA), followed by overnight incubation in developing buffer containing APMA (Life Technologies, Carlsbad, USA) at 37°C. The gel was stained overnight with SYPRO Rudy gel stain (Life Technologies, Carlsbad, USA) at room temperature. The bands of MMPs were visualized by using a LAS-3000 Lumino image analyzer (Fuji Film, Tokyo, Japan). Comparison of the migration level with molecular weight standards (Life Technologies, Carlsbad, USA) and neutralization of MMP-1 by anti-human MMP-1 antibody before loading were performed for identification of MMP-1.

### Statistical analysis

Results of quantitative studies are expressed as means ± SEM. Each data point represents the average of three to twelve independent experiments. Student's t-test was used to compare differences between two groups. One-way ANOVA test was used to make comparisons among three or more groups, and the Tukey-Kramer's post-hoc test was used to identify between group differences. *P* values <0.05 were considered statistically significant.

## Results

### CT-1 induces MMP-1 gene expression

**Figure 1:**
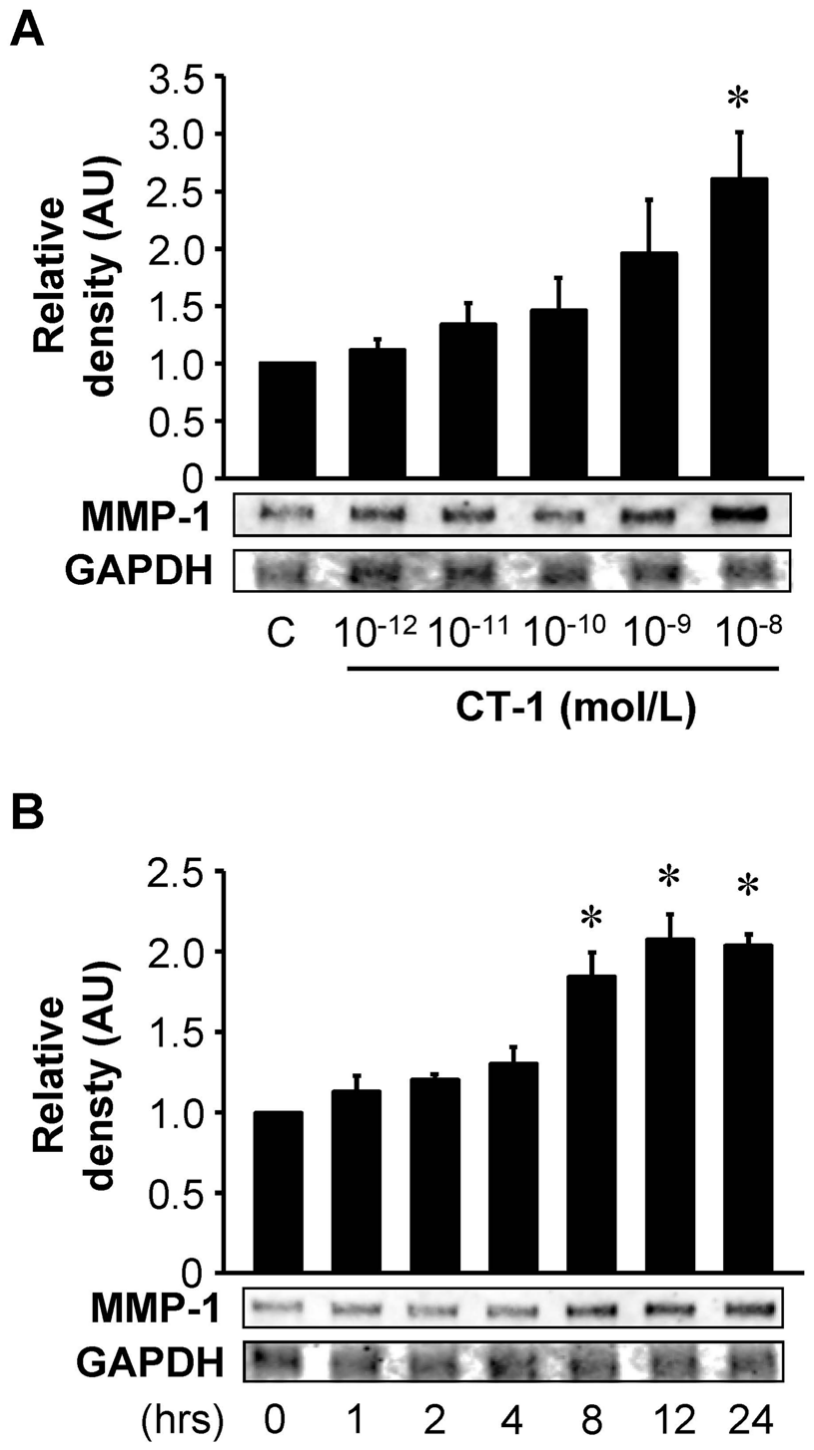
CT-1 stimulates MMP-1 mRNA expression. HAECs were treated for 24 hrs with the indicated concentrations of CT-1 (*A*) or with 10^−8^ mol/L CT-1 for the indicated time (*B*). Total RNA was extracted and subjected to RPA. Results from densitometric analysis are presented as density of MMP-1 mRNA normalized to GAPDH mRNA and relative to control (C) or 0 hr. *P<0.05 vs. C or 0 hr. Blots are representative of 3 independent experiments.

We first investigated whether CT-1 induces MMP-1 mRNA expression in HAECs. HAECs were treated for 24 hours with different doses of CT-1 (10^−12^ to 10^−8^ mol/L) and gene expression of MMP-1 was analyzed by RPA with densitometry. As shown in Fig. 1A, HAECs treated with CT-1 resulted in an increase of MMP-1 mRNA in a dose-dependent manner with a statistical significance at 10^−8^ mol/L. Therefore, unless it is explicitly stated, CT-1 was used at a dose of 10^−8^ mol/L in subsequent experiments. As shown in Fig. 1B, CT-1 increased MMP-1 mRNA in a time-dependent manner with significant increases at 8 to 24 hours of CT-1 treatment.

### CT-1 induces MMP-1 protein expression

**Figure 2:**
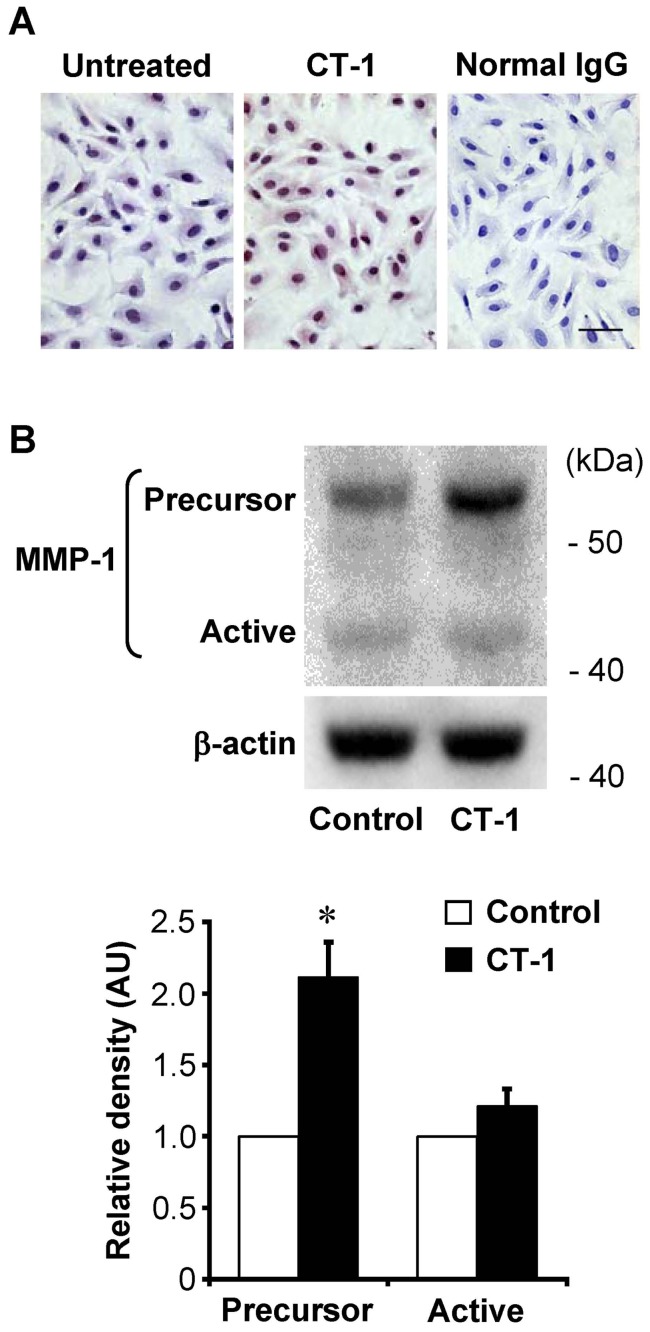
Effect of CT-1 on MMP-1 protein expression. (*A*) Immunocytochemical staining for MMP-1 in HAECs treated with or without 10^−8^ mol/L CT-1 for 24 hrs. Normal mouse IgG served as a negative control. Original magnification; ×400. Bar = 25 μm. (*B*) Western immunoblot analysis of the whole cell lysates using anti-MMP-1 antibodies that recognize both the precursor and the active forms of MMP-1. Bars represent densitometric data of each expression signal after normalization to β-actin and relative to control. *P<0.01 vs. control (*n* = 3).

We then performed immunocytochemical staining and Western immunoblot analysis using anti-MMP-1 antibody that recognizes both precursor and active forms of MMP-1. Immunocytochemistry revealed only weak immunoreactivities of MMP-1 in untreated HAECs, and the staining intensity of MMP-1 was significantly enhanced by treatment with 10^−8^ mol/L CT-1 for 24 hours (Fig. 2A). On the other hand, the cells treated with normal IgG, instead of primary antibody for MMP-1, demonstrated no immnoreactivity. Western immunoblot analysis revealed that HAECs expressed both precursor and active forms of MMP-1 under basal condition, and that the treatment with CT-1 resulted in a significant increase in the precursor form of MMP-1 (Fig. 2B).

### CT-1 induces MMP-1 protein secretion

**Figure 3:**
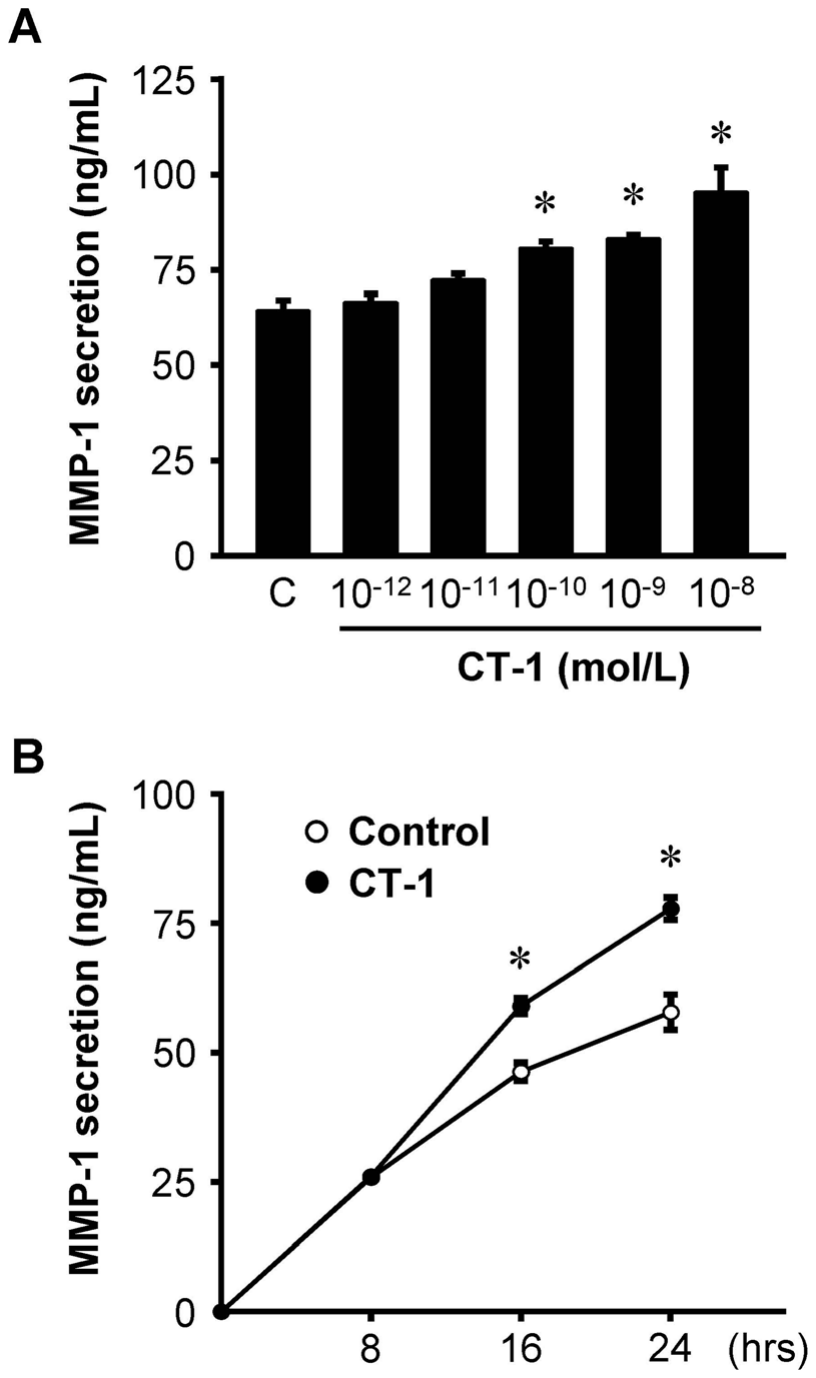
CT-1 stimulates MMP-1 protein secretion. (*A*) MMP-1 secretion in supernatant after treatment with the indicated concentrations of CT-1 for 24 hrs (*n = *12), and (*B*) time course of MMP-1 secretion after treatment with 10^−8^ mol/L CT-1 (*n* = 6). Data represent protein release per 10^5^ cells assayed by ELISA. *P<0.05 vs. control (C) or each control at the same time.

ELISA showed that CT-1 enhanced MMP-1 secretion from HAECs in a dose-dependent manner with significant increases at the doses over 10^−10^ mol/L of CT-1 (Fig. 3A), and that CT-1 induced a time-dependent increase of MMP-1 secretion with statistical significance at 16 and 24 hours of CT-1 treatment (Fig. 3B).

### CT-1-induced MMP-1 secretion is independent of endotoxin, IL-6 and MCP-1 actions

**Figure 4:**
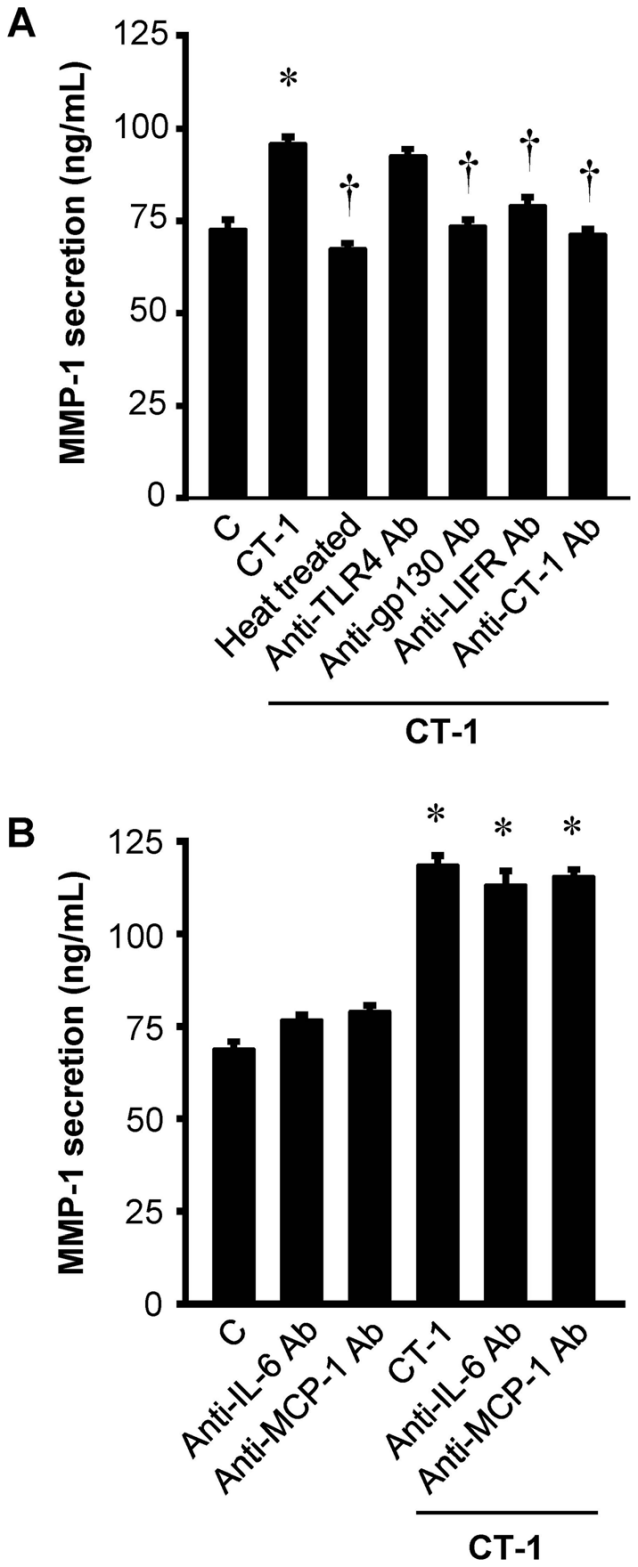
CT-1-induced MMP-1 secretion is independent of endotoxin, IL-6 and MCP-1 actions. (*A*) MMP-1 secretion in supernatant after incubation with heat-treated or untreated 10^−8^ mol/L CT-1 in the presence or absence of neutralizing antibodies against gp130, LIFR or CT-1 as well as TLR4 for 24 hrs. (*B*) Effects of neutralizing antibodies against IL-6 and MCP-1 on basal and CT-1-stimulated MMP-1 secretion from HAECs. Results represent the amount of MMP-1 protein release per 10^5^ cells assayed by ELISA (*n* = 6). *P<0.05 vs. control (C). †*P*<0.05 vs. CT-1.

To exclude the potential artifacts other than the biological actions of CT-1, the following experiments were performed. First, endotoxin concentration in recombinant CT-1 used in the current study is very low (0.074 EU per μg of protein) measured by limulus amebocyte lysate assay (data from PeproTech). Second, neutralizing antibody against TLR4 did not affect CT-1-induced MMP-1 expression at all (Fig. 4A). Third, the enhancement of MMP-1 expression by CT-1 in HAECs was abolished not only by heat treatment (100°C, 30 minutes) but also by the neutralizing antibodies against gp130, LIFR and CT-1 itself (Fig. 4A). Collectively, these findings demonstrated that MMP-1 upregulation observed here is not due to the possible artifact but to the biological actions of CT-1 per se. In addition, neutralizing antibodies against IL-6 and MCP-1, both of which are upregulated by CT-1 and known to stimulate MMP-1 expression [17], [22]–[24], revealed no effects on the CT-1-induced MMP-1 secretion (Fig. 4B).

### CT-1 enhances proteolytic potential in the supernatant of HAECs

**Figure 5:**
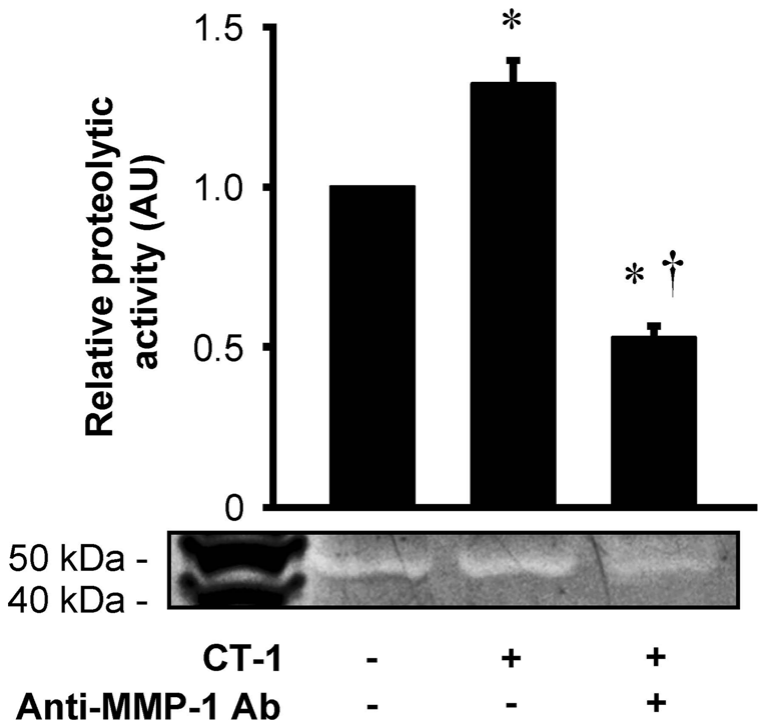
CT-1 enhances proteolytic potential by MMP-1. Supernatant from HAECs with or without stimulation of 10^−8^ mol/L CT-1 for 24 hrs were collected and incubated overnight in the presence or absence of neutralizing antibody against MMP-1 (anti-MMP-1 Ab). They were then run on 12% Tris-Glycine gel with 0.05% casein, and subjected to renaturing, developing and staining procedures. Bars represent results from densitometric analysis of each proteolytic signal relative to control (*n* = 4). *P<0.05 vs. control. †*P*<0.05 vs. CT-1.

Casein zymography showed a proteolytic activity in the supernatant of HAECs as a single molecular weight band, which was significantly enhanced by CT-1 treatment and was significantly reduced by monoclonal neutralizing antibody against MMP-1 (Fig. 5). To clarify the molecular form of MMP-1 in the supernatant of HAECs, MMP-1 activity assay was performed. MMP-1 present in the supernatant of HAECs without CT-1 treatment was predominantly in the precursor form (precursor form, 54 ng/mL/24 hrs; active form, 0.32 ng/mL/24 hrs), and CT-1 significantly increased MMP-1 protein in the precursor form (91 ng/mL/24 hrs, *P*<0.01 vs. without CT-1 treatment), but very little in the active form (0.42 ng/mL/24 hrs). Taken together, the proteolytic activity shown in zymography originated exclusively from the precursor form of MMP-1.

### CT-1 activates JAK/STAT and JNK pathways

**Figure 6:**
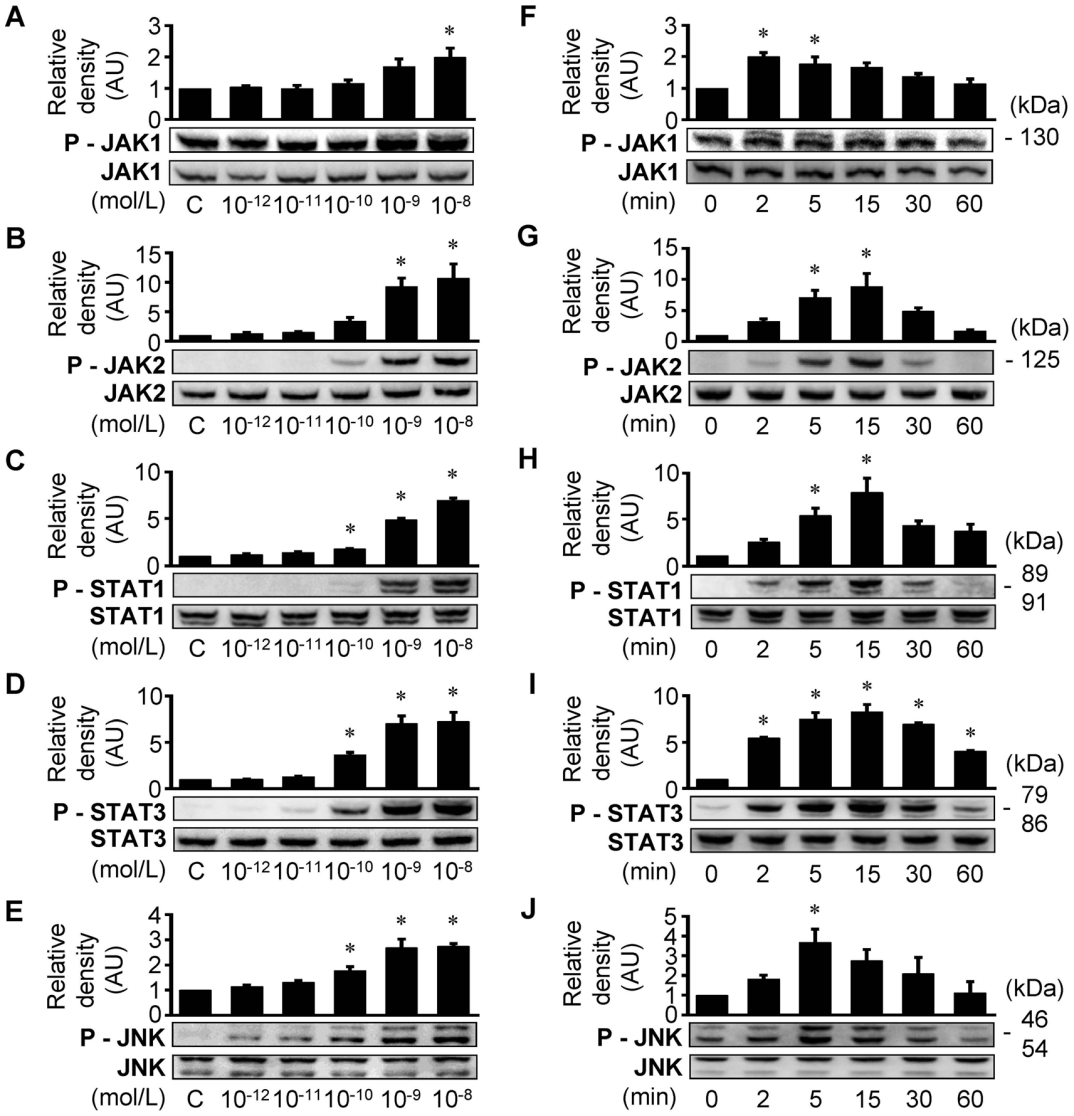
CT-1 activates JAK/STAT cascade and JNK in HAECs. Western immunoblot analysis showing CT-1-stimulated phosphorylation of JAK1 (*A* and *F*), JAK2 (*B* and *G*), STAT1 (*C* and *H*), STAT3 (*D* and *I*) and JNK (*E* and *J*). *A*–*E*: HAECs were treated with 10^−12^ to 10^−8^ mol/L CT-1 for 5 min (*A*) or 15 min (*B*–*E*). *F*–*J*: HAECs were treated with 10^−8^ mol/L CT-1 for 0 to 60 min. Bars represent results from densitometric analysis of each phosphorylation signal after normalization to total protein and relative to control (C) or 0 min. *P<0.05 vs. C or 0 min. Blots are representative of 3 independent experiments.

CT-1 exerts its biological actions through diverse intracellular signal transduction pathways [15]. In our previous studies, we showed that CT-1 phosphorylates extracellular signal-regulated kinase (ERK) 1/2 and p38 MAP kinase in HAECs [17]. In the present study, we additionally confirmed that CT-1 activates JAK/STAT signaling pathway as well as JNK, another member of MAP kinase family, in HAECs. As shown in Fig. 6, CT-1 treatment induced phosphorylation of JAK1, JAK2, STAT1, STAT3, and JNK in a dose- (Fig. 6A–E) and time- (Fig. 6F–J) dependent manner.

### Pharmacological inhibitors of the signaling pathways reduce CT-1-induced MMP-1 gene expression

**Figure 7:**
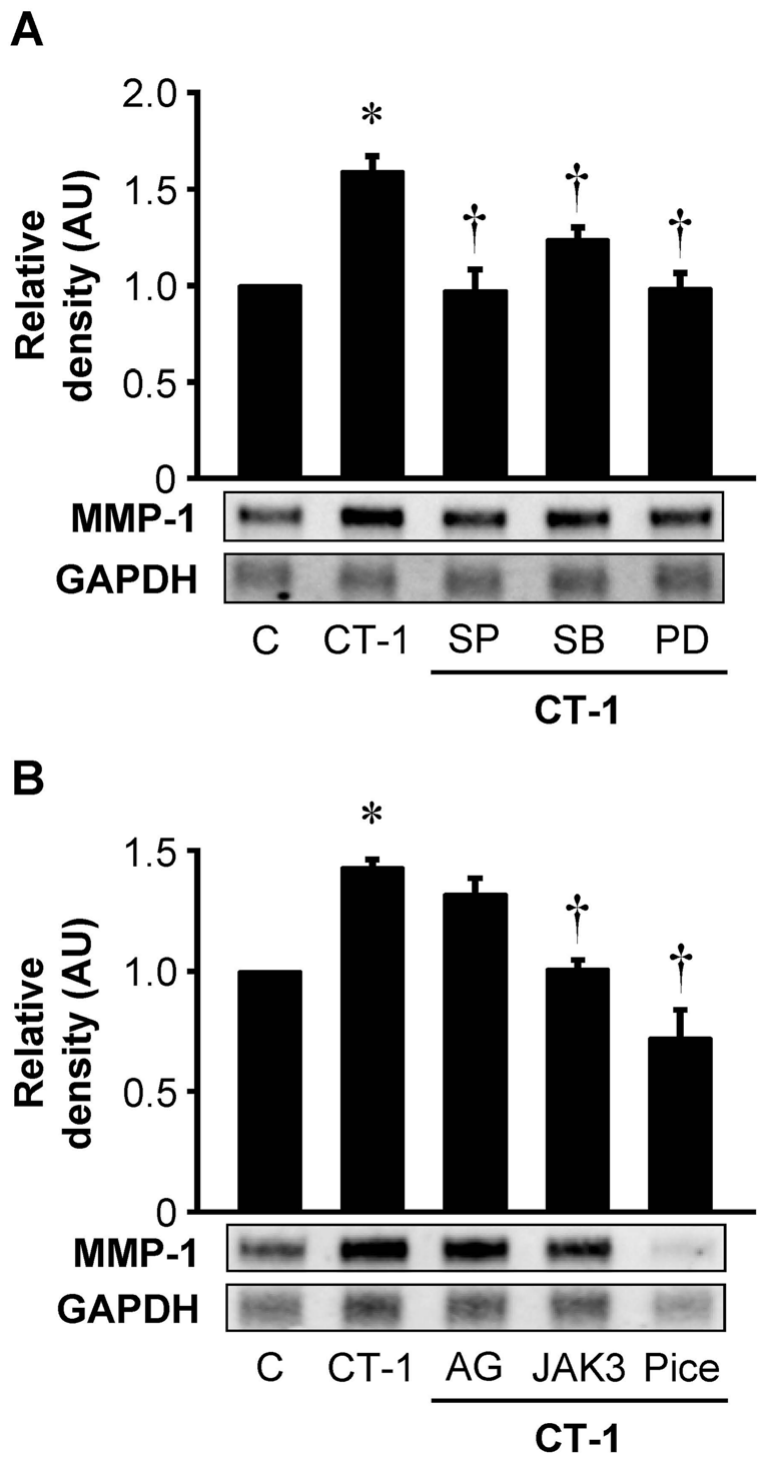
Pharmacological inhibitors of signaling pathways decrease CT-1-induced MMP-1 gene expression. HAECs were preincubated with inhibitors of MAP kinase pathway (*A*) or JAK/STAT cascade (*B*) for 1 hr, followed by additional incubation with 10^−8^ mol/L CT-1 for 24 hrs. Pharmacological inhibitors are 30 μmol/L SP-600125 (SP, JNK inhibitor), 10 μmol/L SB-203580 (SB, p38 MAP kinase inhibitor), 30 μmol/L PD-98059 (PD, ERK1/2 inhibitor), 100 μmol/L AG490 (AG, JAK2 inhibitor), 100 μmol/L JAK3 inhibitor II (JAK3, JAK3 inhibitor) and 100 μmol/L piceatannol (Pice, JAK1 inhibitor). Total RNA was extracted and subjected to RPA. Results from densitometric analysis are presented as density of MMP-1 mRNA normalized to GAPDH mRNA and relative to control (C). *P<0.05 vs. C. †*P*<0.05 vs. CT-1. Blots are representative of 5 (*A*) or 3 (*B*) independent experiments.

We investigated the effects of pharmacological inhibitors of the signaling pathways on CT-1-induced expression of MMP-1 mRNA. HAECs were preincubated with pharmacological inhibitors of MAP kinase pathway including SP-600125 (JNK inhibitor), SB-203580 (p38 MAP kinase inhibitor) and PD-98059 (ERK1/2 inhibitor) for 1 hour and then treated with CT-1 for 24 hours. RPA demonstrated that CT-1-induced augmentation of MMP-1 mRNA expression was significantly attenuated in HAECs pretreated with these inhibitors (Fig. 7A). As shown in Fig. 7B, pharmacological inhibitors of JAK/STAT cascade such as JAK3 inhibitor II (JAK3 inhibitor) and piceatannol (JAK1 inhibitor) also significantly suppressed CT-1-induced expression of MMP-1 mRNA. Although AG490 (JAK2 inhibitor) tended to decrease CT-1-induced MMP-1 mRNA expression, it did not reach statistical significance (Fig. 7B).

### Pharmacological inhibitors of the signaling pathways reduce CT-1-induced MMP-1 protein secretion

**Figure 8:**
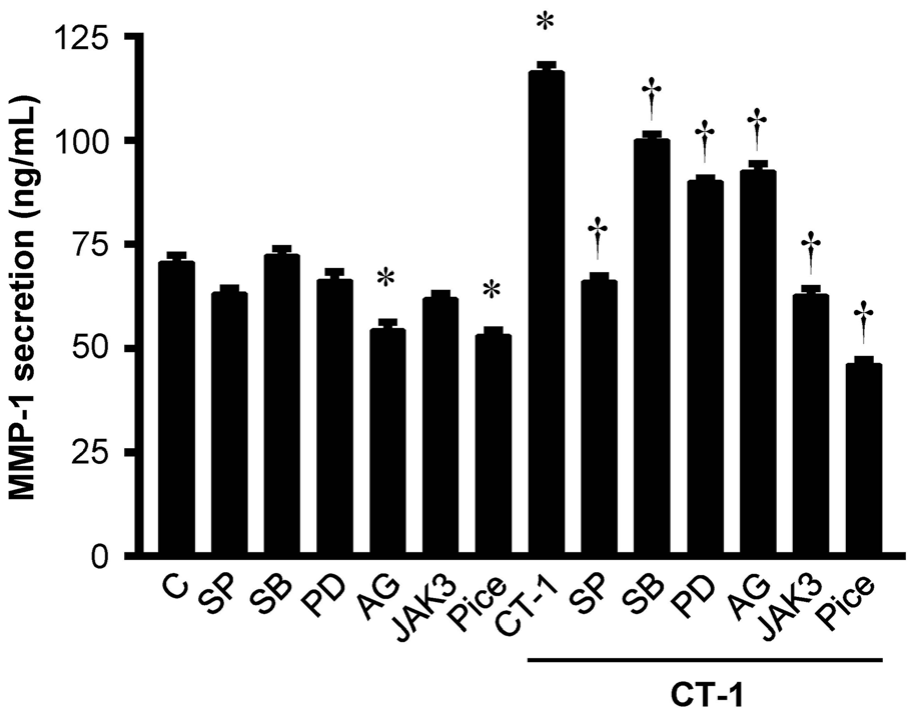
Pharmacological inhibitors of signaling pathways decrease CT-1-induced MMP-1 protein secretion. MMP-1 secretion in supernatant from HAECs with or without stimulation of 10^−8^ mol/L CT-1 for 24 hrs in the presence or absence of pharmacological inhibitors of MAP kinase or JAK/STAT signaling pathways with preincubation for 1 hr. Abbreviations and doses of inhibitors are same as in Figure 7. Results represent the amount of MMP-1 protein release per 10^5^ cells assayed by ELISA (*n* = 6). *P<0.05 vs. control (C). †*P*<0.05 vs. CT-1.

We evaluated basal or CT-1-stimulated MMP-1 protein secretion from HAECs in the presence or absence of pharmacological inhibitors of MAP kinase and JAK/STAT cascades by ELISA. Both AG490 and piceatannol significantly decreased basal MMP-1 secretion from HAECs. All inhibitors such as SP-600125, SB-203580, PD-98059, AG490, JAK3 inhibitor II and piceatannol significantly suppressed CT-1-induced MMP-1 protein secretion from HAECs (Fig. 8).

## Discussion

**Figure 9:**
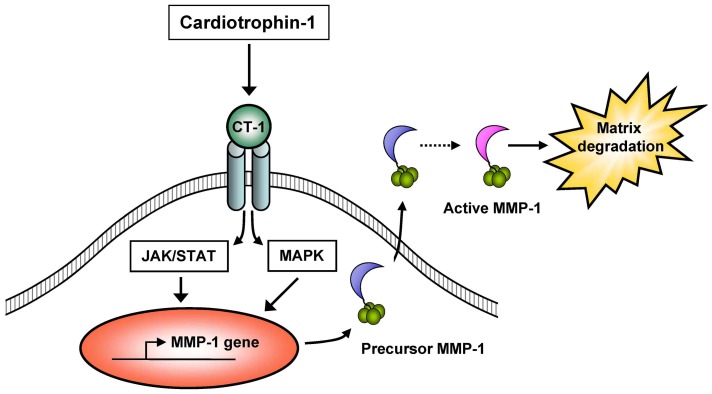
Schematic summary of mechanisms of CT-1-induced MMP-1 expression and proteolytic potential. CT-1 induces MMP-1 gene and protein expression as a source of proteolytic potential via MAP kinase (MAPK) pathway and JAK/STAT cascade in HAECs.

The present study demonstrates for the first time that CT-1 is capable of inducing MMP-1 gene and protein expression via MAP kinase pathway and JAK/STAT cascade in cultured HAECs. In addition, this study also shows that CT-1 stimulates MMP-1 protein synthesis and secretion from HAECs predominantly as a precursor form, which has the potential to be activated and display a proteolytic action (Fig. 9).

MMP-1 has been shown to localize specifically in atheromatous lesions including luminal endothelial cells, but weakly or not at all in non-atherosclerotic arteries [6]. In the present study, immunocytochemistry revealed only weak immunoreactivities of MMP-1 in untreated HAECs, which were significantly enhanced by the treatment of CT-1 (Fig. 2A). RPA and Western immunoblot analysis also showed that MMP-1 mRNA and protein expressions in HAECs were detected even under basal conditions, and increased by CT-1 stimulation (Fig. 1 and Fig. 2B). Furthermore, CT-1 enhanced MMP-1 protein secretion from HAECs in a dose- and time-dependent manner (Fig. 3). In a previous investigation, we showed that immunoreactivity for CT-1 and its receptor components are colocalized in human atherosclerotic tissues [17]. This immunohistochemical localization of CT-1 is similar to the distribution of augmented MMP-1 in human atherosclerotic plaques [6], [7]. Taken together, it is suggested that CT-1 might upregulate MMP-1 expression not only in cultured HAECs but also in the atherosclerotic plaques *in vivo*.

Other members of IL-6 cytokine family to which CT-1 belongs, such as oncostatin M and IL-6, have already been shown to be implicated in the regulation of ECM degradation by upregulating MMPs in vascular cells [19], [25], [26]. In the current study, we demonstrated CT-1-induced MMP-1 upregulation in HAECs. However, in contrast to these findings, Weiss et al. [27] reported that IL-6 family including CT-1, oncostatin M, IL-6 and leukemia inhibitory factor has no effects on the expression of MMP-1 in human adult cardiac myocytes and fibroblasts. In addition, CT-1 has been shown to stimulate canine cardiac fibroblast growth [28]. Recently, Lopez-Andres et al. [29] also demonstrated that CT-1 increases the secretion of collagen type III and elastin, and the expression of tissue inhibitors of metalloproteinases (TIMPs) without altering metalloproteinase activity in vascular smooth muscle cells of spontaneously hypertensive rats. Although these reports are contradictory to the results of this study, the reason for this discrepancy remains unclear. Different cell types studied, presence or absence of serum deprivation and different experimental procedures including incubation periods could be considered. Alternatively, CT-1 may exert dual actions in HAECs that stimulate either plaque stabilizing or plaque destabilizing process, depending on the vascular situations and circumstances [8].

It should be noted that the CT-1-induced MMP-1 expression described here may be associated with the indirect effects of CT-1 via local induction of cytokines or chemokines which subsequently stimulate MMP-1 expression in HAECs [30]. For example, IL-6 and MCP-1 have been shown to be upregulated by CT-1 in vascular cells, and these factors have also been reported to stimulate MMP-1 expression [17], [22]–[24]. To examine whether these factors are involved in CT-1-mediated MMP-1 upregulation, further studies using neutralizing antibodies were performed. The neutralizing antibodies against IL-6 and MCP-1 revealed no effect on the CT-1-induced MMP-1 secretion (Fig. 4B). This means that neither IL-6 nor MCP-1 mediate the MMP-1 upregulation by CT-1 treatment.

We are interested in whether the regulatory effect of CT-1 on MMP-1 involves its enzymatic activity. MMP-1 activity assay demonstrated that CT-1 stimulates MMP-1 protein secretion from HAECs predominantly as the precursor form, and that this precursor form is not changed to the active form at least for 24 hrs of treatment with CT-1, suggesting that CT-1 itself has no direct effect to activate MMP-1. However, zymography using supernatant of HAECs showed a proteolytic potential attributed to MMP-1 after APMA treatment, indicating that CT-1-induced precursor form of MMP-1 could be functionally activated after secretion and display a proteolytic action. It is well known that almost all MMPs are originally secreted as the precursor forms and then activated through multiple pathways involving other MMPs, proteinases and other factors [3].

To elucidate the signaling pathways by which CT-1 induces gene and protein expressions of MMP-1 in HAECs, we investigated the major signaling pathways for IL-6 type cytokine family, such as MAP kinase pathway and JAK/STAT cascade [31]. In addition to our previous study showing that CT-1 phosphorylates ERK 1/2 and p38 MAP kinase in HAECs [17], we here confirmed that CT-1 induces phosphorylation of JAK1, JAK2, STAT1, STAT3, and JNK in a dose- and time-dependent manner (Fig. 6). Furthermore, pharmacological inhibitor studies indicated the important roles of the MAP kinase pathway and JAK/STAT cascade in mediating CT-1-induced MMP-1 expression (Fig. 7 and Fig. 8). Although there are many papers proving the role of MAP kinase pathway in the regulation of MMP-1 expression in various cell types [10], [32], [33], the role of JAK/STAT cascade have not been fully investigated. In support of our results, Aida et al. [22] recently demonstrated that IL-6 and soluble IL-6 receptor stimulate the production of MMPs via not only ERK1/2 MAP kinase pathway but also JAK/STAT cascade in human chondrocytes. Interestingly, the inhibitory effects of AG490 (JAK2 inhibitor) on gene and protein expression of MMP-1 were not so definitive compared with those of JAK3 inhibitor II (JAK3 inhibitor) and piceatannol (JAK1 inhibitor), implying that CT-1 could trigger induction of MMP-1 more dominantly through JAK1 and JAK3 than JAK2 in HAECs (Fig. 7 and Fig. 8). The same tendency has been shown in a few reports concerning the signaling pathways that induce MMPs expression [34], [35].

One of the limitations in the present study is that we used relatively high concentrations of CT-1 compared with the plasma level. But the exact concentration of CT-1 in vascular tissue remains unknown to date. Because CT-1 is mainly produced in the heart and also in the vasculature, we speculate that local CT-1 concentration in coronary arteries, especially at atherosclerotic lesions, might be much higher than that in the peripheral plasma. Another limitation is that we narrowly focused our interest to the effect of CT-1 on MMP-1 expression in HAECs, and evaluated neither other MMPs nor TIMPs in the current study. Since the extracellular matrix metabolism depends on the local balance between collagens, MMPs, TIMPs and other proteases such as stromelysin or plasmin [24], the observations *in vitro* reported here do not necessarily reflect conditions in vascular endothelium or atheroma *in vivo*. Yet, the previous report showing that plasma concentration of CT is elevated in patients with unstable angina compared with those with stable angina supports our hypothesis that CT-1 induces MMP-1 in vascular endothelium and contributes to the plaque instability in patients with unstable angina [18]. Therefore, systemic administration of CT-1 in the treatment of cardiac diseases such as acute coronary syndrome should be considered carefully [36], [37]. Further studies are necessary to elucidate the actual effect of CT-1 on the plaque instability.

In conclusion, the present study demonstrates that CT-1 stimulates MMP-1 expression and its proteolytic potential through ERK1/2, p38 MAP kinase, JNK and JAK/STAT pathways. In light of our findings, we propose that CT-1 may play a critical role in the pathophysiology of plaque instability leading to acute coronary syndrome.

## References

[1] Libby P (2002) Inflammation in atherosclerosis. Nature 420: 868–874. 12490960 10.1038/nature01323

[2] Libby P (1995) Molecular bases of the acute coronary syndromes. Circulation 91: 2844–2850. 7758192 10.1161/01.cir.91.11.2844

[3] Dollery CM, Libby P (2006) Atherosclerosis and proteinase activation. Cardiovasc Res 69: 625–635. 16376322 10.1016/j.cardiores.2005.11.003

[4] Newby AC (2008) Metalloproteinase expression in monocytes and macrophages and its relationship to atherosclerotic plaque instability. Arterioscler Thromb Vasc Biol 28: 2108–2114. 18772495 10.1161/ATVBAHA.108.173898

[5] Stary HC, Chandler AB, Dinsmore RE, Fuster V, Glagov S, et al (1995) A definition of advanced types of atherosclerotic lesions and a histological classification of atherosclerosis. A report from the Committee on Vascular Lesions of the Council on Arteriosclerosis, American Heart Association. Circulation 92: 1355–1374. 7648691 10.1161/01.cir.92.5.1355

[6] Galis ZS, Sukhova GK, Lark MW, Libby P (1994) Increased expression of matrix metalloproteinases and matrix degrading activity in vulnerable regions of human atherosclerotic plaques. J Clin Invest 94: 2493–2503. 7989608 10.1172/JCI117619 PMC330083

[7] Sukhova GK, Schonbeck U, Rabkin E, Schoen FJ, Poole AR, et al (1999) Evidence for increased collagenolysis by interstitial collagenases-1 and -3 in vulnerable human atheromatous plaques. Circulation 99: 2503–2509. 10330380 10.1161/01.cir.99.19.2503

[8] Browatzki M, Larsen D, Pfeiffer CA, Gehrke SG, Schmidt J, et al (2005) Angiotensin II stimulates matrix metalloproteinase secretion in human vascular smooth muscle cells via nuclear factor-kappaB and activator protein 1 in a redox-sensitive manner. J Vasc Res 42: 415–423. 16103692 10.1159/000087451

[9] Hanemaaijer R, Koolwijk P, le Clercq L, de Vree WJ, van Hinsbergh VW (1993) Regulation of matrix metalloproteinase expression in human vein and microvascular endothelial cells. Effects of tumour necrosis factor alpha, interleukin 1 and phorbol ester. Biochem J 296 ( Pt 3): 803–809. 10.1042/bj2960803 PMC1137766 8280080

[10] Montero I, Orbe J, Varo N, Beloqui O, Monreal JI, et al (2006) C-reactive protein induces matrix metalloproteinase-1 and -10 in human endothelial cells: implications for clinical and subclinical atherosclerosis. J Am Coll Cardiol 47: 1369–1378. 16580524 10.1016/j.jacc.2005.10.070

[11] Wang H, Keiser JA (1998) Vascular endothelial growth factor upregulates the expression of matrix metalloproteinases in vascular smooth muscle cells: role of flt-1. Circ Res 83: 832–840. 9776730 10.1161/01.res.83.8.832

[12] Kishimoto T, Taga T, Akira S (1994) Cytokine signal transduction. Cell 76: 253–262. 8293462 10.1016/0092-8674(94)90333-6

[13] Kunisada K, Hirota H, Fujio Y, Matsui H, Tani Y, et al (1996) Activation of JAK-STAT and MAP kinases by leukemia inhibitory factor through gp130 in cardiac myocytes. Circulation 94: 2626–2632. 8921810 10.1161/01.cir.94.10.2626

[14] Demyanets S, Huber K, Wojta J (2012) Vascular effects of glycoprotein130 ligands – part I: pathophysiological role. Vascul Pharmacol 56: 34–46. 22197898 10.1016/j.vph.2011.12.004

[15] Jougasaki M (2010) Cardiotrophin-1 in cardiovascular regulation. Adv Clin Chem 52: 41–76. 21275339 10.1016/s0065-2423(10)52002-x

[16] Jougasaki M, Larsen AM, Cataliotti A, Christiansen DC, Burnett JC Jr (2002) Cardiotrophin-1 stimulates endothelin-1 via gp130 in vascular endothelial cells. Peptides 23: 1441–1447. 12182945 10.1016/s0196-9781(02)00078-5

[17] Ichiki T, Jougasaki M, Setoguchi M, Imamura J, Nakashima H, et al (2008) Cardiotrophin-1 stimulates intercellular adhesion molecule-1 and monocyte chemoattractant protein-1 in human aortic endothelial cells. Am J Physiol Heart Circ Physiol 294: H750–763. 18055523 10.1152/ajpheart.00161.2007

[18] Talwar S, Squire IB, Downie PF, Davies JE, ng LL (2000) Plasma N terminal pro-brain natriuretic peptide and cardiotrophin 1 are raised in unstable angina. Heart 84: 421–424. 10995414 10.1136/heart.84.4.421 PMC1729429

[19] Zhu Y, Hojo Y, Ikeda U, Takahashi M, Shimada K (2000) Interaction between monocytes and vascular smooth muscle cells enhances matrix metalloproteinase-1 production. J Cardiovasc Pharmacol 36: 152–161. 10942155 10.1097/00005344-200008000-00003

[20] Jougasaki M, Schirger JA, Simari RD, Burnett JC Jr (1998) Autocrine role for the endothelin-B receptor in the secretion of adrenomedullin. Hypertension 32: 917–922. 9822453 10.1161/01.hyp.32.5.917

[21] Laragione T, Shah A, Gulko PS (2012) The vitamin D receptor regulates rheumatoid arthritis synovial fibroblast invasion and morphology. Mol Med 18: 194–200. 22064970 10.2119/molmed.2011.00410 PMC3320133

[22] Aida Y, Honda K, Tanigawa S, Nakayama G, Matsumura H, et al (2012) IL-6 and soluble IL-6 receptor stimulate the production of MMPs and their inhibitors via JAK-STAT and ERK-MAPK signalling in human chondrocytes. Cell Biol Int 36: 367–376. 22087578 10.1042/CBI20110150

[23] Fritzenwanger M, Meusel K, Foerster M, Kuethe F, Krack A, et al (2006) Cardiotrophin-1 induces interleukin-6 synthesis in human umbilical vein endothelial cells. Cytokine 36: 101–106. 17197193 10.1016/j.cyto.2006.10.015

[24] Yamamoto T, Eckes B, Mauch C, Hartmann K, Krieg T (2000) Monocyte chemoattractant protein-1 enhances gene expression and synthesis of matrix metalloproteinase-1 in human fibroblasts by an autocrine IL-1 alpha loop. J Immunol 164: 6174–6179. 10843667 10.4049/jimmunol.164.12.6174

[25] Nagata T, Kai H, Shibata R, Koga M, Yoshimura A, et al (2003) Oncostatin M, an interleukin-6 family cytokine, upregulates matrix metalloproteinase-9 through the mitogen-activated protein kinase kinase-extracellular signal-regulated kinase pathway in cultured smooth muscle cells. Arterioscler Thromb Vasc Biol 23: 588–593. 12615664 10.1161/01.ATV.0000060891.31516.24

[26] Sakata K, Hamaoka K, Ozawa S, Niboshi A, Yahata T, et al (2010) Matrix metalloproteinase-9 in vascular lesions and endothelial regulation in Kawasaki disease. Circ J 74: 1670–1675. 20534943 10.1253/circj.cj-09-0980

[27] Weiss TW, Kvakan H, Kaun C, Zorn G, Speidl WS, et al (2005) The gp130 ligand oncostatin M regulates tissue inhibitor of metalloproteinases-1 through ERK1/2 and p38 in human adult cardiac myocytes and in human adult cardiac fibroblasts: a possible role for the gp130/gp130 ligand system in the modulation of extracellular matrix degradation in the human heart. J Mol Cell Cardiol 39: 545–551. 15890357 10.1016/j.yjmcc.2005.03.015

[28] Tsuruda T, Jougasaki M, Boerrigter G, Huntley BK, Chen HH, et al (2002) Cardiotrophin-1 stimulation of cardiac fibroblast growth: roles for glycoprotein 130/leukemia inhibitory factor receptor and the endothelin type A receptor. Circ Res 90: 128–134. 11834704 10.1161/hh0202.103613

[29] Lopez-Andres N, Fortuno MA, Diez J, Zannad F, Lacolley P, et al (2010) Vascular effects of cardiotrophin-1: a role in hypertension? J Hypertens 28: 1261–1272. 20216087 10.1097/HJH.0b013e328337fe42

[30] Galis ZS, Muszynski M, Sukhova GK, Simon-Morrissey E, Unemori EN, et al (1994) Cytokine-stimulated human vascular smooth muscle cells synthesize a complement of enzymes required for extracellular matrix digestion. Circ Res 75: 181–189. 8013077 10.1161/01.res.75.1.181

[31] Heinrich PC, Behrmann I, Haan S, Hermanns HM, Muller-Newen G, et al (2003) Principles of interleukin (IL)-6-type cytokine signalling and its regulation. Biochem J 374: 1–20. 12773095 10.1042/BJ20030407 PMC1223585

[32] Cortez DM, Feldman MD, Mummidi S, Valente AJ, Steffensen B, et al (2007) IL-17 stimulates MMP-1 expression in primary human cardiac fibroblasts via p38 MAPK- and ERK1/2-dependent C/EBP-beta , NF-kappaB, and AP-1 activation. Am J Physiol Heart Circ Physiol 293: H3356–3365. 17921324 10.1152/ajpheart.00928.2007

[33] Turner NA, Warburton P, O'Regan DJ, Ball SG, Porter KE (2010) Modulatory effect of interleukin-1alpha on expression of structural matrix proteins, MMPs and TIMPs in human cardiac myofibroblasts: role of p38 MAP kinase. Matrix Biol 29: 613–620. 20619343 10.1016/j.matbio.2010.06.007 PMC3004031

[34] Kim S, Kim Y, Lee Y, Chung JH (2008) Ceramide accelerates ultraviolet-induced MMP-1 expression through JAK1/STAT-1 pathway in cultured human dermal fibroblasts. J Lipid Res 49: 2571–2581. 18664717 10.1194/jlr.M800112-JLR200

[35] Li WQ, Dehnade F, Zafarullah M (2001) Oncostatin M-induced matrix metalloproteinase and tissue inhibitor of metalloproteinase-3 genes expression in chondrocytes requires Janus kinase/STAT signaling pathway. J Immunol 166: 3491–3498. 11207308 10.4049/jimmunol.166.5.3491

[36] Liao Z, Brar BK, Cai Q, Stephanou A, O'Leary RM, et al (2002) Cardiotrophin-1 (CT-1) can protect the adult heart from injury when added both prior to ischaemia and at reperfusion. Cardiovasc Res 53: 902–910. 11922900 10.1016/s0008-6363(01)00531-4

[37] Ruixing Y, Dezhai Y, Jiaquan L (2004) Effects of cardiotrophin-1 on hemodynamics and cardiomyocyte apoptosis in rats with acute myocardial infarction. J Med Invest 51: 29–37. 15000253 10.2152/jmi.51.29

